# An innovative charge‐based extracellular vesicle isolation method for highly efficient extraction of EV‐miRNAs from liquid samples: miRQuick

**DOI:** 10.1002/jex2.126

**Published:** 2023-12-08

**Authors:** Junsoo Park, Minju Bae, Hyeonah Seong, Jin hwa Hong, Su Jin Kang, Kyung hwa Park, Sehyun Shin

**Affiliations:** ^1^ Department of Micro‐Nano Engineering Korea University Seoul South Korea; ^2^ Engineering Research Center for Biofluid Biopsy Seoul South Korea; ^3^ School of Mechanical Engineering Korea University Seoul South Korea; ^4^ Division of Oncology/Hematology, College of Medicine Korea University Seoul South Korea; ^5^ Department of Bioengineering and Nano‐Bioengineering Incheon National University Incheon South Korea

**Keywords:** EV, ExoPAS, isolation, miRNAs, miRQuick

## Abstract

Extracellular vesicle‐derived microRNAs (EV‐miRNAs) are promising biomarkers for early cancer diagnosis. However, existing EV‐miRNA extraction technologies have a complex two‐step process that results in low extraction efficiency and inconsistent results. This study aimed to develop and evaluate a new single‐step extraction method, called miRQuick, for efficient and high‐recovery extraction of EV‐miRNAs from samples. The miRQuick method involves adding positively charged substances to the sample, causing negatively charged EVs to quickly aggregate and precipitate. A membrane lysate is then added to extract only miRNA. The entire process can be completed within an hour using standard laboratory equipment. We validated the miRQuick method using various analytical techniques and compared its performance to other methods for plasma, urine and saliva samples. The miRQuick method demonstrated significantly higher performance than other methods, not only for blood plasma but also for urine and saliva samples. Furthermore, we successfully extracted and detected nine biomarker candidate miRNAs in the plasma of breast cancer patients using miRQuick. Our results demonstrate that miRQuick is a rapid and efficient method for EV‐miRNA extraction with excellent repeatability, making it suitable for various applications including cancer diagnosis.

## 1 INTRODUCTION

MicroRNAs (miRNAs) are short, non‐coding RNAs that play a critical role in regulating gene expression (Bartel, 2004). They modulate post‐transcriptional gene expression by cleaving target mRNA molecules, inhibiting translation or controlling mRNA turnover through base‐pairing with target mRNA molecules (Ameres & Zamore, 2013; Davey et al., 2021; Filipowicz et al., 2008). Additionally, miRNAs have been identified in various body fluids and are recognized as significant cancer biomarkers (Kosaka et al., 2010; Thind & Wilson, 2016; Weber et al., 2010), with potential for non‐invasive molecular diagnosis (Davey et al., 2021; Min et al., 2019; Ostenfeld et al., 2016; Thind & Wilson, 2016; Weber et al., 2010).

Extracellular vesicles (EVs) are small vesicles secreted by cells, ranging in size from 50 to 1000 nm (Raposo & Stoorvogel, 2013). These vesicles play a crucial role in protecting various nucleic acids and proteins, including miRNAs, from degradation by in vivo enzymes (Cheng et al., 2014; Ge et al., 2014). However, obtaining stable EV‐miRNA from biological fluids is challenging due to RNase degradation in these fluids (Andreu et al., 2016; Liu et al., 2019), and isolating EVs from samples is complicated due to their small size and the presence of protein and lipid components in liquid samples (Raposo & Stoorvogel, 2013; Zhang et al., 2018). Although ultracentrifugation (UC) is the gold standard for EV isolation, it is labour‐intensive, time‐consuming and can potentially damage EVs due to high g forces used during separation (Jeppesen et al., 2014). To address these limitations, various technologies based on size, affinity, nanomaterials, fluid chips and polymers have been developed for efficient EV separation (Contreras‐Naranjo et al., 2017; Dao et al., 2022; Deregibus et al., 2016; Kang et al., 2022, Kim & Shin, 2021; Kim et al., 2020; Pammi Guru et al., 2022). For instance, the methodology of protamine‐based EV isolation has been previously described in our previous studies (Kim & Shin, 2021; Kim et al., 2020) and others (Deregibus et al., 2016).

Current methodologies for the isolation of EV‐associated miRNA face significant limitations that hinder their suitability for both clinical and research applications. These limitations include low recovery rates and operational inconveniences. The prevailing approach involves a two‐step process, initiating with EV isolation followed by miRNA extraction, often resulting in very poor performance due to the application of mismatched techniques. Our study was meticulously crafted with a profound understanding of the challenges encountered by research facilities when executing these two steps independently. These challenges encompass issues such as low yield, extended operation times and the potential for cross‐contamination.

In light of these challenges, there exists an urgent demand for an efficient EV‐miRNA extraction technology suitable for molecular diagnosis in clinical settings. While a few commercial products have emerged to address this unmet need, they typically commence by introducing a lysis buffer into the sample to disrupt exosome membranes. Subsequently, they facilitate the precipitation and removal of proteins, ultimately enabling miRNA extraction. In contrast, our study introduces a novel EV‐miRNA extraction technology that involves EV precipitation through charge‐based clustering using cationic salt, followed by miRNA extraction utilizing a spin‐column method. This pioneering approach holds immense potential for advancing liquid biopsy techniques in cancer and other diseases by enabling the efficient extraction of EV‐associated miRNA from human liquid samples. Our research significantly would contribute to the overarching goal of developing a clinically applicable EV‐miRNA extraction technology, which, in turn, promises to enhance molecular diagnostic capabilities.

## 2 MATERIAL AND METHODS

### 2.1 Operating principles

**FIGURE 1:**
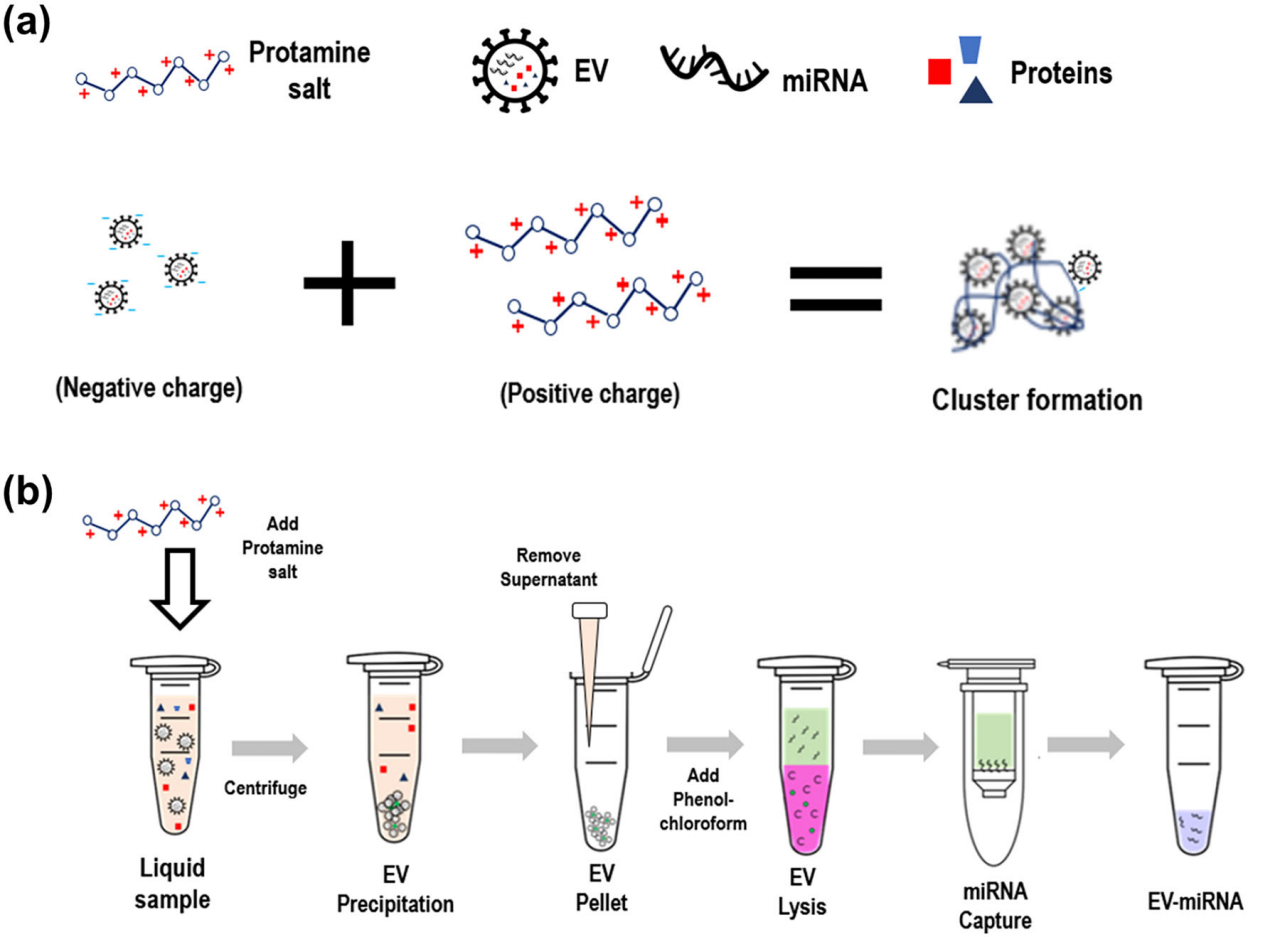
Schematic of charge‐based EV precipitation and spin‐column method for miRNA extraction (miRQuick). (a) Cluster formation by protamine salt (PS) with EVs using ExoPAS. (b) Experimental procedure to extract miRNA from a liquid sample using the spin column method.

The present study introduces an innovative platform called miRQuick for the isolation of EV‐miRNA from biofluids, incorporating a charge‐based EV cluster precipitation (ExoPAS) and spin column method. To isolate EVs from liquid samples, the ExoPAS method employed protamine sulphate salt (Sigma‐Aldrich, Burlington, MA, USA), which induces the formation of clusters with negatively charged EVs upon addition to the sample (Figure 1). Specifically, the protamine salt (PS) is dissolved in DI water and added to the liquid sample, which is then incubated at 4°C for 30 min. After incubation, the sample is centrifuged at 3000 × *g* for 30 min, and the supernatant is removed with a pipette.

Next, to lyse the EV pellet, a phenol‐based lysis buffer was added, and the mixture was incubated at room temperature for 5 min. After lysis, 90 µL of chloroform was added and vortexed to separate the phenol‐based lysis buffer and chloroform into distinct phases, with miRNAs present in the chloroform layer. The mixture was then centrifuged at 12,000 × *g* for 15 min, and the aqueous layer on top was collected from the phase‐separated solution. The solution obtained after chloroform extraction was mixed with twice the volume of pure ethanol. Subsequently, the mixture was transferred to a spin column and centrifuged. The silica membrane‐bound miRNAs were washed with 700 µL of washing buffer 1 and 500 µL of washing buffer 2 twice. After the washing step, the spin column was dried by centrifuging at 10,000 × *g* for 5 min in a 2‐mL collection tube, followed by transfer to a fresh 1.5‐mL elution tube. For the elution step, 30 µL of elution buffer was added to the centre of the spin column and centrifuged at 10,000 × *g* for 1 min to elute the EV‐miRNA.

### 2.2 Sample preparation (blood, saliva, and urine)

This study was conducted in accordance with the principles of the Declaration of Helsinki. The study procedure was approved by the Institutional Review Board of Korea University Anam Hospital in Seoul, Republic of Korea (IRB project number: 2016AN0090). With informed consent, blood samples (3 mL) were collected from participants using K2‐EDTA vacutainers (Becton Dickinson, Franklin Lakes, NJ, USA). The whole blood was centrifuged at 1900 *g* for 10 min using a model 1248 device (LABOGEN, Denmark) to separate the plasma, which was further centrifuged at 12,000 *g* for 15 min to remove any large particles. Urine and saliva samples were collected in sterile containers and then purified by centrifugation. Both samples were centrifuged at 3000 *g* for 15 min to precipitate and remove large particles. The recovered supernatant was then filtered using a mesh with pores of 800 nm to remove particles larger than 800 nm. The samples obtained in this way were divided into 1 mL each and stored. Unless otherwise specified, the volume of all samples in this study was fixed at 1 mL.

### 2.3 EV isolation using ExoPAS

The efficiency of EV isolation is crucial for extracting EV‐miRNA. We developed an innovative platform for isolating EV isolation (ExoPAS), which is a charge‐based EV cluster precipitation. The present method was compared with UC and two commercially available kits: PEG‐based precipitation (ExoQuick) and spin column‐based (exoEasy).

#### 2.3.1 EV isolation using ultracentrifuge

Although UC is a labour‐intensive, repetitive and time‐consuming procedure (taking more than 6 h), it has been considered the gold standard method for EV separation. In this study, plasma was mixed with PBS (phosphate buffered saline) in a 1:3 ratio to separate EVs by UC. Centrifugation at 3000 × *g* for 15 min and at 12,000 × *g* for 30 min was sequentially performed to remove large vesicles such as cell debris from the mixed sample. The supernatant was then centrifuged for 2 h at 120,000 × *g* and 4°C using a high‐speed centrifuge (CP100WX; Hitachi, Tokyo, Japan). After removal of the supernatant, the pellet at the bottom was resuspended and washed in PBS at 120,000 *g* and 4°C for 1 h and then finally resuspended in 200 µL of PBS.

#### 2.3.2 PEG‐based EV isolation

For PEG (polyethylene glycol) based EV isolation, ExoQuick exosome precipitation solution (EXOQ5A‐1; System Biosciences, Palo Alto, CA, USA) was applied. PEG‐based ExoQuick solution was mixed with the plasma sample, and the resulting mixture was then incubated for 30 min at 4°C. After incubation, the mixture was carefully centrifuged at 1500 × *g* for 30 min, removing the supernatant while leaving the pellet in the tube. All traces of the ExoQuick solution were removed after a second round of centrifugation at 1500 × *g* for 5 min, and then the pellet was resuspended in 200 µL of PBS.

#### 2.3.3 Spin column‐based EV isolation

EV from plasma was isolated using the exoEasy™ Maxi Kit from Qiagen in Valencia, California, USA, as directed by the manufacturer. Briefly, plasma filtration was done to remove particles larger than 0.8 m in diameter. After adding an equal volume of XBP buffer and thoroughly combining it, the suspension was added to an exoEasy spin column and centrifuged for 1 min at 500 × *g*. The flow‐through was removed. Following the addition of 10 mL of XWP buffer, the buffer was centrifuged at 5000 × *g* for 5 min. Following this, the flow‐through was removed, the spin column was moved to a new collection tube, 200 µL of XE buffer was added, the mixture was centrifuged for 5 min at 5000 × *g* and the flow‐through was collected. The final collected solution is EV.

### 2.4 miRNA extraction by commercial products

We utilized two kits for miRNA extraction in this study. The protocols for both kits were followed as provided by the manufacturers.

The first kit used was the Nucleospin kit (Macherey‐Nagel, Germany), a commercial product for single‐step miRNA extraction from plasma. Initially, 290 µL of MLP buffer was mixed with 1‐mL plasma and incubated for 3 min at room temperature. Then, 90 µL of MPP buffer was added and the mixture was centrifuged at 11,000 × *g* for 3 min. The supernatant was collected and mixed with 400 µL isopropanol. The resulting sample was then loaded onto a spin column and centrifuged at 11,000 × *g* for 30 s at room temperature. The column was then washed with 600‐µL MW1 buffer and centrifuged at 11,000 × *g* for 30 s. The wash was repeated with 700‐µL MW1 buffer followed by 250‐µL MW2 buffer and centrifugation at 11,000 × *g* for 30 s. The columns were centrifuged at 11,000 × *g* for another 2 min to remove all the ethanol. Finally, the miRNA was eluted in 30‐µL RNase‐free water.

The second kit used was the exoRNeasy kit (QIAGEN, USA), a two‐step kit for miRNA extraction. In brief, 1‐mL plasma per specimen was mixed with 1 volume of binding buffer and added to the exoEasy spin column. After centrifugation (500 × *g*, 1 min), the flow‐through was discarded and wash buffer was added to the spin column. After another centrifugation (5000 × *g*, 5 min), and discarding of the flow‐through, the vesicles were lysed by adding 700‐µL QIAzol to the spin column, and the lysate was collected by centrifugation (5000 × *g*, 5 min, rt). Following the addition of 90‐µL chloroform, thorough mixing and centrifugation (12,000 × *g*, 5 min, 4°C) to separate organic and aqueous phases, the aqueous phase was recovered and mixed with 2 volumes of 100% ethanol. The sample‐ethanol mixture was added to an RNeasy MinElute spin column and centrifuged (8500 × *g*, 15 s). The column was washed once with buffer RWT (8500 × *g*, 15 s), and then twice with buffer RPE (8500 × *g*, 15 s and 2 min) followed by membrane drying (12,000 × *g*, 5 min) and elution of RNA into nuclease‐free water (12,000 × *g*, 1 min). This procedure allowed concentrating the extracellular RNA from 1‐mL plasma into a final volume of 30 µL of water.

### 2.5 Preparation of Trizol buffer

Trizol was used to lysis EVs. We created Trizol based on previous research (Zepeda & Verdonk, 2022). All components of Lysis buffer (phenol, guanidine thiocyanate, ammonium thiocyanate, glycerol, sodium acetate) were purchased from sigma (Burlington, MA, USA). Trizol lysis buffer consists of 0.4‐M ammonium thiocyanate, 0.8‐M guanidine thiocyanate, 0.1‐M sodium acetate (pH 5.2), 5% (v/v) glycerol and 38% (v/v) phenol. Phenol was dissolved in a ∼60°C water bath and all other chemical reagents were dissolved in RNase‐free water. The Trizol buffer was stored at 4°C.

### 2.6 Zeta potential analyser

EV, cluster and PS zeta potentials were measured using a zeta potential analyser (Zetasizer Pro; Malvern Panalytical, Malvern, UK). Since it is difficult to resuspend the EV cluster in deionized water, 990 µL of deionized water was added after suspension in 10‐µL NaCl solution. PS was coated and measured on silica nanoparticles for zeta potential analysis.

### 2.7 Scanning electron microscopy (SEM) Images

An anodic aluminium oxide (AAO) membrane mounted in a gasket was used for SEM measurements of EVs and EV clusters isolated from plasma, urine and saliva. Samples for SEM measurement were filtered through a membrane. After filtering, these membranes were incubated in glutaraldehyde solution (Sigma‐Aldrich, St. Louis, MO, USA) for 30 min. The membranes were sequentially rinsed with 25, 50, 75, 90 and 100% ethanol and incubated 2 h at 37°C in a dry oven. After coating the membrane with Pt, EVs and clusters subjects existing on the membranes were observed by SEM (Quanta 250 FEG; FEI, Hillsboro, OR, USA).

### 2.8 Nanoparticle tracking analysis (NTA)

NTA was performed using a NS300 and NTA 3.4 Software (NanoSight, Wiltshire, UK). EV samples were diluted in filtered PBS. Three 30‐s videos were recorded of each sample with camera level 14 and detection threshold set at 11. Video images of the EVs were acquired, and their mean size and concentration were analysed according to each dilution factor.

### 2.9 Western blot assay

Proteins analysed in the Western blot assay were extracted from EVs suspended in 200 µL. Proteins extracted from EV were mixed with Laemmli buffer and 2‐mercaptoethanol (BioRad, USA) and heated at 95°C for 10 min. SDS‐PAGE Mini‐PROTEAN® TGX™ Precast Gel (Bio‐rad, USA) was used to separate protein samples by gel electrophoresis. For western blotting, CD9, CD81, TSG101, ALIX, which are EV‐specific markers, and albumin were used as negative marker, referring to MISEV2018 (Théry et al., 2018). Recombinant anti‐CD9 antibody (ab92726, Abcam, Cambridge, UK), recombinant anti‐CD81 antibody (ab109201, Abcam, Cambridge, UK), recombinant anti‐TSG101 antibody (ab125011, Abcam, Cambridge, UK), recombinant anti‐ALIX antibody (ab186429, Abcam, Cambridge, UK) and goat anti‐rabbit IgG H&L were used for immunoblotting after that (ab205718, Abcam, Cambridge, UK). ChemiDoc™ XRS + System (Bio‐Rad, CA, USA) was used to examine the protein bands using enhanced chemiluminescence (ECL) reagent following antibody incubation.

### 2.10 Quantification of RNA

Isolated RNA concentration and purity ratio measurement using Nanodrop DS‐11 FX+ spectrophotometer (Denovix, Wilmington, DE, USA). RT‐qPCR for confirming EV‐derived miRNA concentration used hsa‐Let‐7a and hsa‐miR‐142 as miRNAs known to exist in EV (Blondal et al., 2013). To quantify EV‐derived miRNA, the RNA eluate was reverse transcribed using the TaqMan™ MicroRNA RT Kit (4366596, Life Technologies, Eugene, OR, USA) and TaqMan MicroRNA Assay (4427975, Life Technologies, Eugene, OR, USA). After that, has‐let‐7a‐5p (ID 000377) and has‐miR‐142‐3p (ID000464) were measured using both the miRNA and TaqMan Universal Master Mix II without UNG (4440040, Life Technologies, Eugene, OR, USA). As demonstrated in Figure S1, it was verified that the quantity of miRNA extracted from EVs remained consistent, unaffected by storage temperature or the storage duration of EVs.

### 2.11 RNA extraction and RT‐qPCR

RNA was isolated from plasma samples using miRQuick (present method). The stability of the miRQuick reagent used in this study was confirmed to yield consistent results for up to 1 year, even when stored at room temperature. This is evident from the accelerated aging test results displayed in Figure S2 as well as Table S1. RNA concentration and purity were measured using Nanodrop DS‐11 FX+ spectrophotometer (Denovix, Wilmington, DE, USA). In total, 25 ng of RNA was revers‐transcribed with the TaqMan™ MicroRNA RT Kit (4366596, Life Technologies, Eugene, OR, USA) and TaqMan MicroRNA Assay (4427975, Life Technologies, Eugene, OR, USA). Then, miR‐93‐5p, miR‐24‐3p, miR‐4529‐3p, miR‐21‐5p, miR‐6875‐5p, miR‐223‐5p, miR‐373‐3p, miR‐3616‐3p and miR‐1245 analysed quantitative PCR with TaqMan Universal Master Mix II without UNG (4440040, Life Technologies, Eugene, OR, USA).

All data were analysed using GraphPad Prism 7 (GraphPad Software, LA Jolla, CA, USA). Significant differences between the groups were assessed using an unpaired parametric two‐tailed *t*‐test assuming equal standard deviations. Samples were considered statistically significant when the *p* value was lower than 0.05.

### 2.12 Cellular uptake assay

To confirm the uptake of EV by the HDF cell line, EV was stained with PKH67 green dye (Sigma‐Aldrich, Burlington, MA, USA) for 15 min at 25°C. The mixture was filtrated using a 100‐kDa filter to remove free PKH67 dye. HDF cells were cultured in a medium with PKH‐labelled EV at a concentration of 2 × 109 particles/mL. For nuclear staining, Hoechst 33342 dye was added to the culture medium. Cellular uptake of EV was analysed using a fluorescence microscope (Nikon, Tokyo, Japan).

### 2.13 Cytotoxicity test

To measure the cell viability, cells were seeded in 96‐well plates for 24 h. After washing, cells were incubated with EV‐depleted FBS containing medium added EV at a concentration of 2 × 109 particles/mL for 72 h. Cell viability was measured by a WST‐1 assay.

## 3 RESULTS

### 3.1 Cationic salt‐induced EV cluster formation: ExoPAS

**FIGURE 2:**
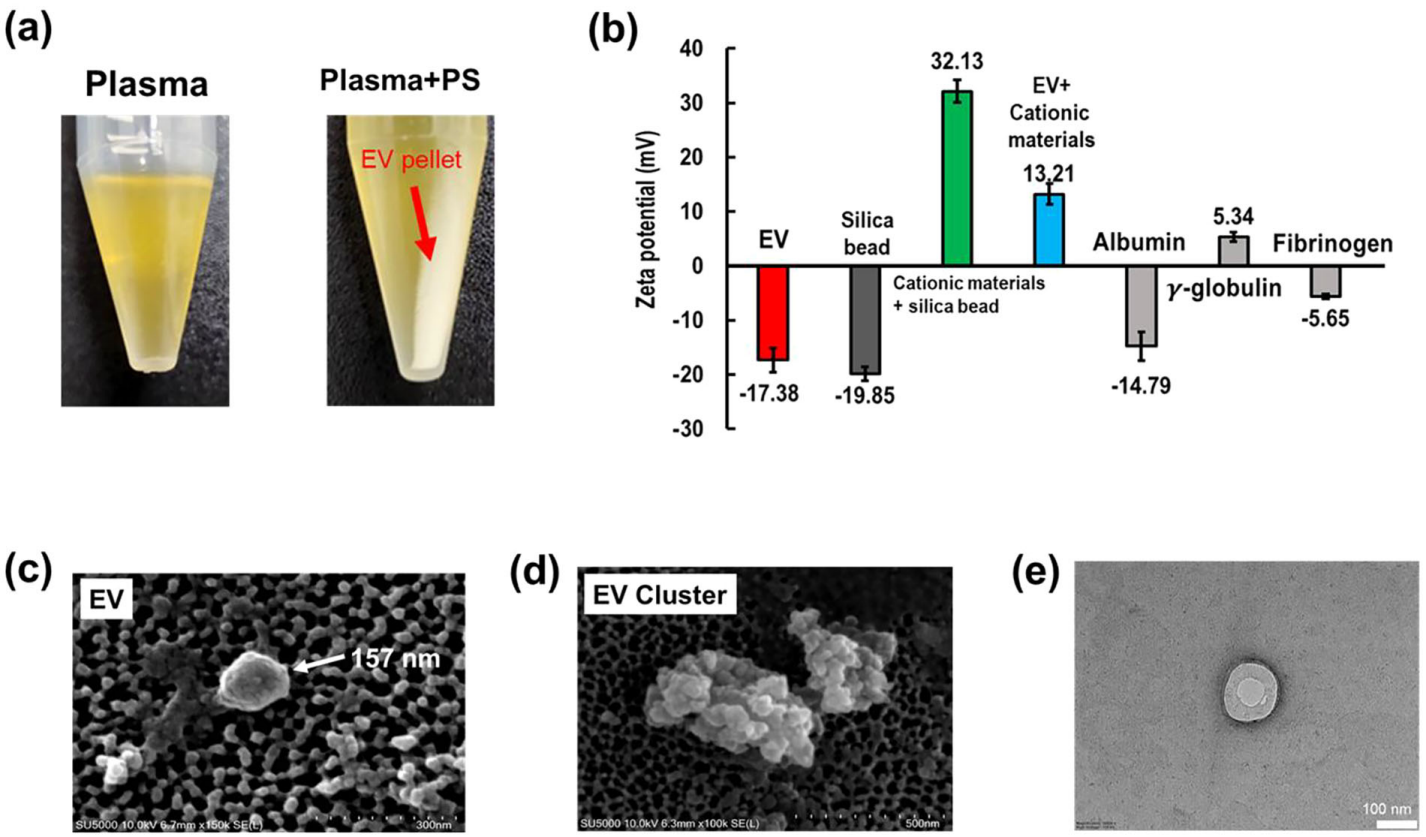
Charge‐based EV clusters with ExoPAS. (a) EV pellet formation by adding protamine salt (PS) to plasma and centrifuging, (b) Zeta potential of EV, Silica bead, protamine salt + silica bead, EV + protamine salt, albumin, γ‐globulin and fibrinogen, (*n* = 3); (c) SEM image of isolated EVs using protamine salt precipitation from plasma sample, (d) SEM image of EV clustering formation with cationic protamine salt, (e) TEM image of an isolated EV using ExoPAS.

To confirm the formation of charge‐based clusters between EVs and PS, we conducted an experiment where a mixture of plasma and PS was incubated at 4°C for 30 min and then centrifuged. As shown in Figure 2a, pellets were not formed in the tube containing only plasma after centrifugation. However, EV‐PS cluster (EV‐PSC) pellets were observed in the mixture. In addition, incubation of the sample and PS mixture was carried out at 4°C for 30 min. The detailed results are shown in Figure S1a,b. We also measured the zeta potential to confirm the electrostatic interaction between EVs and PS (Figure 2b). The zeta potential measurements show that EVs have a negative surface charge of −17.4 mV, while PS‐coated silica beads have a positive charge of +32.1 mV, and EV‐PSCs of ExoPAS method have a zeta potential of 13.2 mV, indicating a strong electrostatic interaction between the negatively charged EVs and positively charged PS. In addition, proteins that are abundant in plasma also are negatively charged. The zeta potential measurements show that albumin, globulin and fibrinogen have a negative surface charge of −16.94, +4.09 and −5.11 mV, respectively. This indicates that EV‐PSCs may contain a variety of plasma proteins. To reduce the presence of proteins, desalting or affinity resin‐type spin columns can be used. In the current downstream protocol, miRNA extraction processes using phenol–chloroform can be employed to remove proteins. SEM was used to characterize EVs. NaCl 1‐M solution was used to resuspend the EV‐PSC pellets. In a previous study, it was confirmed that ion exchange was successful in NaCl ‐M solution (Kim & Shin, 2021). The size of isolated EV was about 157 nm, and the formation of EV clusters was observed (Figure 2c,d). Furthermore, Figure S3 displays SEM images of EVs isolated from a plasma sample using ExoPAS. Particles displaying shapes distinct from the spherical structure, as indicated by the red arrow, are presumed to be non‐EVs. Figure 2e showcases the TEM image of an EV isolated from a plasma sample. Supplementary TEM images of isolated EVs from saliva and urine samples can be found in Figure S4.

### 3.2 Isolation EV from urine and saliva samples

**FIGURE 3:**
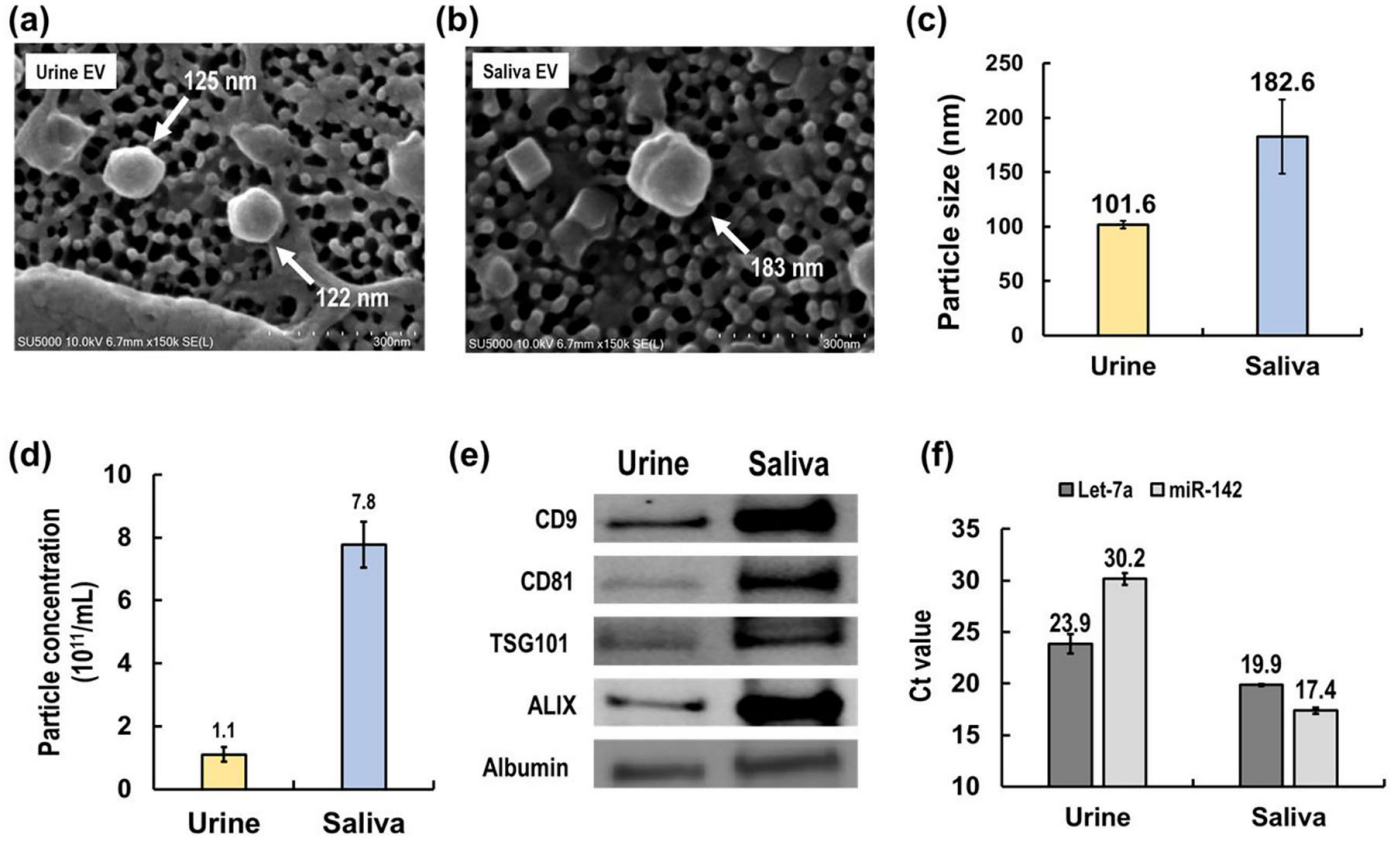
Characterization of isolated EVs from urine and saliva samples using ExoPAS. (a) Scanning electron microscopy (SEM) image of isolated EVs from a urine sample. (b) SEM image of isolated EVs from a saliva sample. (c) Mean size of isolated EVs from urine and saliva measured by nanoparticle tracking analysis (NTA). (d) Particle concentrations of EVs isolated from urine and saliva measured by NTA. (e) Western blot analysis for protein markers of isolated EVs from urine and saliva. (f) EV‐derived miRNAs (hsa‐let‐7a‐5p, hsa‐miR‐142‐3p) measured by reverse transcription‐quantitative polymerase chain reaction (RT‐qPCR).

We isolated EVs from urine and saliva to confirm that the EV‐PSCs of miRQuick method can be applied to various samples. SEM, NTA, Western blot and RT‐qPCR were used to confirm isolated EVs. As shown in Figure 3a,b, EVs isolated using EV‐PSC from urine and saliva sample were characterized by SEM. EVs isolated from urine sample were measured at 122, 125 nm, and EVs isolated from saliva sample were measured at 183 nm. In addition, as a result of NTA analysis, the average size of EVs isolated from urine was 101.6 nm, and EVs isolated from saliva were 182.6 nm, which was larger than urine EVs (Figure 3c).

The EV concentration was 1.1 × 10^11^ in urine and 7.8 × 10^11^ in saliva, confirming that saliva isolated about seven times as many as EVs (Figure 3d). The concentration of EVs can vary depending on the collection conditions of the samples. Therefore, it is difficult to ascertain which samples, urine or saliva, are rich in EVs. For further validation of isolated EVs, protein and miRNA markers were confirmed by Western blot and RT‐qPCR analysis. Exosome‐specific markers (CD9, CD81, TSG101, ALIX) showed clear bands in both EVs isolated from the samples (Figure 3e). The whole Western blot corresponding to Figure 3 is available in Figure S5. NTA identified sharper bands in saliva samples with higher EV concentrations. Saliva samples with higher concentrations of EVs had more miRNAs (Figure 3f). Unusually, let‐7a showed a higher concentration in the trampled sample, and miR‐142 showed a higher concentration in the saliva sample. These results confirm that the EV‐PSC of miRQuick can be effectively applied to a range of bodily fluids.

### 3.3 Comparison of EV isolation from plasma

**FIGURE 4:**
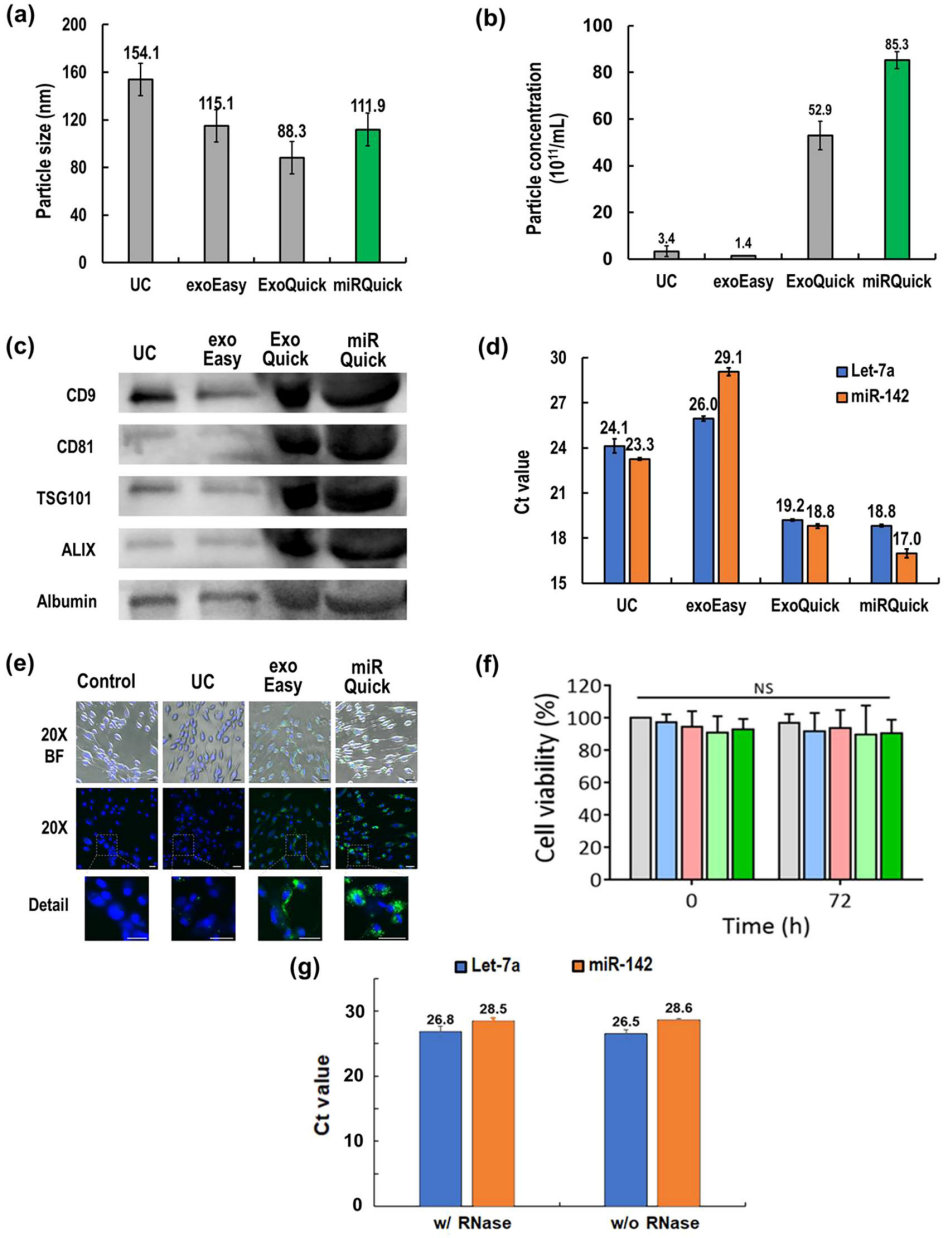
Comparison of EV isolation methods (EQ, UC, exoEasy, miRQuick using NTA, western blot, RT‐qPCR). (a) Particle size, (b) particle concentration, (c) Western blot for protein markers of EVs. (d) EV‐derived miRNAs (has‐let‐7a‐5p, has‐miR‐142‐3p) measured by RT‐qPCR, (e) Cellular uptake of EVs into HDF. Green and blue colours indicate EVs and nuclei, respectively (scale bar: 50 µm). (f) Cytotoxicity test of EVs in HDF, (g) miRNA RT‐qPCR results with and without RNase.

NTA, western blot and RT‐qPCR were performed to confirm EV isolation efficiency. NTA analysis was used to confirm the size distribution (40–160 nm) and concentration of the isolated EVs. The EVs were also subjected to western blot analysis to verify their surface and internal proteins, while RT‐qPCR was used to confirm the concentrations of EV‐miRNAs. As shown in Figure 4a, the sizes of the EVs isolated by the four different methods were ranged from 88 to 154 nm, which were within the expected range. However, there were significant differences in EV concentrations between the isolation methods. The EV‐PSC of miRQuick method, which uses PS, showed the highest yield (85.3 × 10^11^/mL), as indicated in Figure 4b. This was 60 times higher than the yield of exoEasy (1.4 × 10^11^/mL).

The PEG‐based precipitation method also showed high yields, while UC, considered the gold standard, had similar yields to exoEasy. In other words, the clustering and precipitating methods (ExoQuick and miRQuick) led to the best EV recovery rate among others.

We used a western blot assay (Figure 4c) to identify surface and internal protein markers of EVs. ExoQuick and miRQuick showed a thick band, indicating a high number of EVs. All isolation methods (UC, exoEasy, ExoQuick, miRQuick) showed relatively clear bands for the internal factor CD9. However, the internal markers TSG101 and ALIX, as well as the surface marker CD81, showed faint bands in UC and exoEasy, which have low EV recovery rates. We also confirmed the presence of albumin, which is abundant in plasma, in all EV isolation methods. The bands isolated by ExoQuick and EV‐PSC of miRQuick appeared bent compared to other methods, which may indicate the presence of coagulation factors in the isolated EVs. It can be interpreted that ExoQuick and EV‐PSC separate a large number of EVs, but also separate other foreign substances present in plasma. Previous studies have shown that precipitation‐based EV isolation technology has the highest yield but the lowest purity. Thus, ExoQuick and EV‐PSC of miRQuick were found to have high EV recovery rates but also other substances present in plasma. These findings should be taken into account when selecting an EV isolation method for downstream applications. The whole Western blot corresponding to Figure 4 is available in Figure S6.

RT‐qPCR analysis (Figure 4d) was performed to identify EV‐miRNAs. The results were consistent with the NTA analysis. The miRQuick method yielded the most EV‐miRNAs (Ct value: hsa‐let‐7a: 18.8, hsa‐miR‐142: 17.0), followed by ExoQuick (Ct value: hsa‐let‐7a: 19.2, hsa‐miR‐142: 18.8), UC (Ct value: hsa‐let‐7a: 24.1, hsa‐miR‐142: 23.3) and exoEasy (Ct value: hsa‐let‐7a: 26.0, hsa‐miR‐142: 29.1). Notably, the concentration of miR‐142 was higher than that of let‐7a in EV‐derived miRNA isolated using most methods, except exoEasy, where let‐7a was higher than miR‐142. These results suggest that the EV isolation methods may affect the EVsʼ contents. Furthermore, we confirmed that the miRQuick method has superior separation efficiency from plasma compared to other methods.

We further tested the delivery of EVs into human cells because EVs exert their biological activities through interaction with and internalization by cells. To confirm this, human dermal fibroblasts (HDF) were supplemented with EVs stained with PKH67 green dye at a concentration of 2.0 × 109 particles/mL and incubated for 6 h. As shown in Figure 4e, fluorescence microscopy revealed that a high number of EVs penetrated into the cells and mainly localized in the cytoplasm. The results demonstrated the robust intracellular delivery of EV regardless of the isolation method used. That is, the EVs isolated using miRQuick (PS method) also maintain their biological properties.

Cytotoxicity of EVs was tested using EVs isolated by each method. As shown in Figure 4f, EVs prepared by each isolation method, including UC, exoEasy and miRQuick, exhibited no discernible toxicity when exposed to HDF. These results strongly suggest the safety and biocompatibility of EVs derived from miRQuick. Additionally, after precipitating EVs with PS, the pellet was resuspended in distilled water. RNase treatment was applied to the resuspended solution, and then lysis and RNase inactivation using Trizol. Subsequently, miRNA extraction was carried out using a spin column with the same process as miRQuick. The Ct values of miR‐142 and let‐7a showed consistent results regardless of the presence of RNase, as shown in Figure 4g.

### 3.4 Comparison of EV‐miRNA extraction from plasma

**FIGURE 5:**
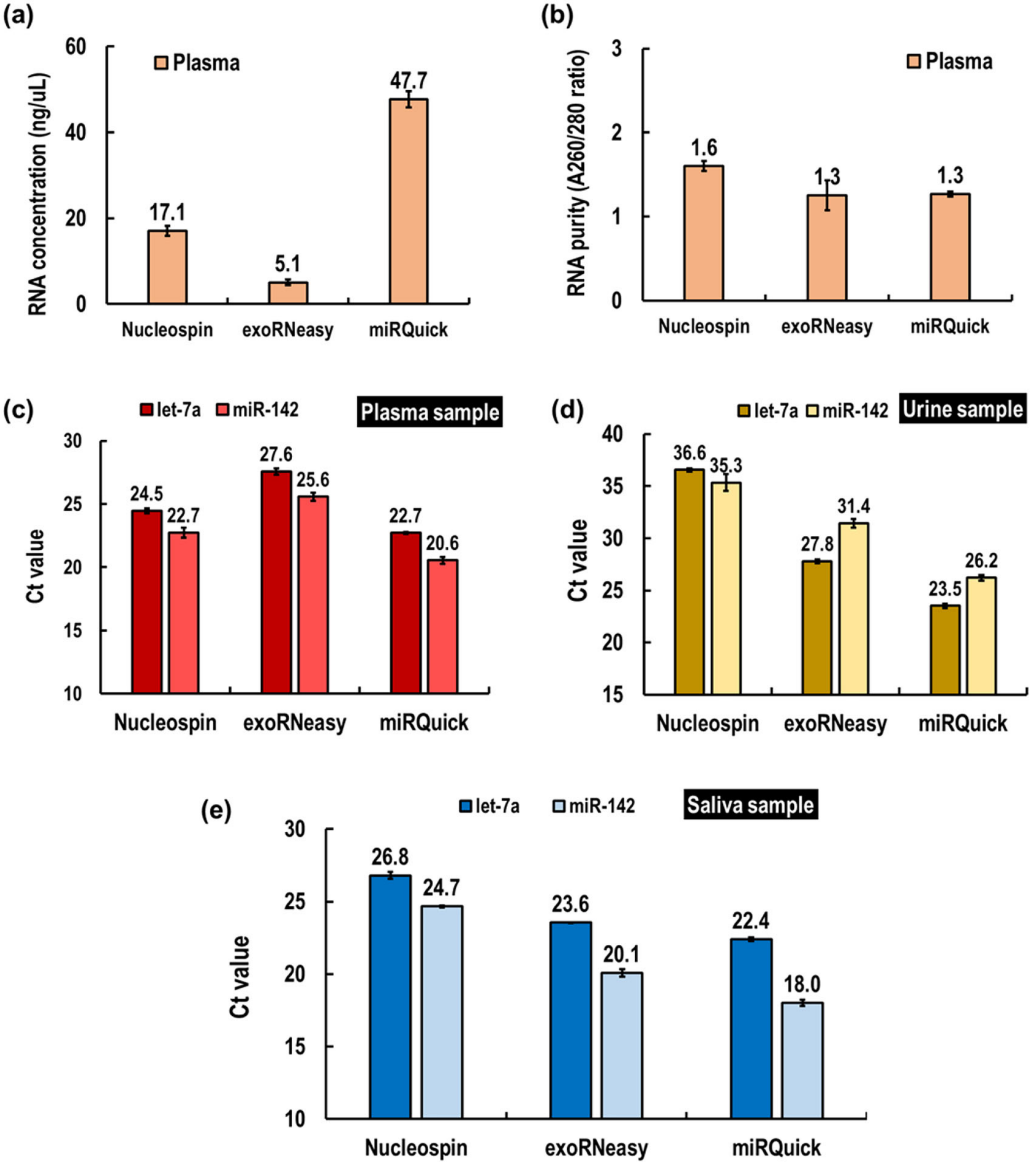
Comparison of miRNA extraction commercial kits (Nucleospin, miRNeasy) with miRQuick using Nanodrop and RT‐qPCR. (a) Measurement RNA concentration by nanodrop, (b) measurement RNA purity ratio by nanodrop. (c) EV‐derived miRNAs (has‐let‐7a‐5p, has‐miR‐142‐3p) measured by RT‐qPCR from a plasma sample. (d) EV‐derived miRNAs (has‐let‐7a‐5p, has‐miR‐142‐3p) measured by RT‐qPCR from a urine sample. (e) EV‐derived miRNAs (has‐let‐7a‐5p, has‐miR‐142‐3p) measured by RT‐qPCR from a saliva sample.

A comparative analysis was conducted to evaluate miRQuick against other commercially available kits, namely NucleoSpin miRNA and exoRNeasy, for miRNA extraction from different sample types (Figure 5). Saliva samples yielded the highest RNA extraction yields across all methods, followed by urine and plasma (Figure 5a). Specifically, NucleoSpin recovered 115.7 ng/mL of RNA from saliva, miRNeasy recovered 101.0 ng/mL and miRQuick recovered 239.4 ng/mL. In urine, NucleoSpin recovered 50.8 ng/mL, miRNeasy recovered 86.8 ng/mL and miRQuick recovered 159.8 ng/mL of RNA. From plasma, NucleoSpin recovered 17.1 ng/mL, miRNeasy recovered 5.1 ng/mL and miRQuick recovered 47.7 ng/mL of RNA. Notably, phenol–chloroform‐based RNA extraction has been reported to yield higher RNA amounts (2.4–93 times) compared to general silica column‐based extraction methods (Deng et al., 2005; Santiago‐Vázquez et al., 2006; Xiang et al., 2001). However, caution must be exercised while handling phenol due to its carcinogenic properties, and automation can be challenging due to supernatant separation requirements. The purity of the extracted RNA was assessed by A260/280 ratio (Figure 5b), with values of 1.5–2.0 generally considered as indicative of pure RNA (Wilfinger et al., 1997). In our study, RNA extracted using all three methods showed purity levels ranging from 1.3 to 1.8. Thus, our proposed miRQuick method demonstrated the highest efficiency and cost‐effectiveness in extracting high‐purity RNA, surpassing all existing methods, including commercial kits.

The extraction performance of EV‐miRNAs for the three different methods was further compared and analysed by RT‐qPCR analysis (Figure 5c–e). The target miRNAs were carefully selected with Let‐7a and miR‐142 considered as housekeeping genes in EVs. We adopted the stem‐loop primer method that specifically synthesizes only miRNA into cDNA for RT‐qPCR (Chen et al., 2005). In plasma samples, miRNA concentrations were found to be highest with miRQuick (Let‐7a: 20.6, miR‐142: 22.7), followed by NucleoSpin (Let‐7a: 24.5, miR‐142: 22.7) and exoRNeasy (Let‐7a: 27.6, miR‐142: 25.6) (Figure 5c). Similarly, in urine samples, miRQuick (Let‐7a: 23.5, miR‐142: 26.2) showed the lowest Ct values, followed by exoRNeasy (Let‐7a: 27.8, miR‐142: 31.4) and NucleoSpin (Let‐7a: 36.6, miR‐142: 35.3) (Figure 5d). Lastly, in saliva samples, miRNA concentrations were highest with exoRQuick (Let‐7a: 18.0, miR‐142: 22.4), followed by exoRNeasy (Let‐7a: 23.6, miR‐142: 20.1), and NucleoSpin (Let‐7a: 26.8, miR‐142: 24.7) (Figure 5e). Our results confirmed that the miRQuick method is effective in extracting miRNAs from plasma, urine and saliva samples. miRQuick utilizes a cationic material to precipitate EVs based on charge and enables miRNA extraction after EV separation, making it a versatile option for various liquid samples. Furthermore, we anticipate that miRQuick may be applicable for EV‐miRNA extraction from other liquid samples such as cerebrospinal fluid, milk, fruit juice and more.

### 3.5 Evaluation of cancer‐related miRNAs between healthy groups and breast cancer patients

To evaluate the clinical applicability of our method, we conducted a mini‐scale clinical study to investigate the diagnostic potential of EV‐derived miRNAs isolated from both healthy individuals (*n* = 5) and breast cancer (BC) patients (*n* = 5). Through a comprehensive review of relevant literature (Jang et al., 2021; Shimomura et al., 2016; Yoshikawa et al., 2018; Zou et al., 2021), we carefully selected seven potential BC biomarkers, namely miR‐93‐5p, miR‐24‐3p, miR‐1246, miR‐21‐5p, miR‐6875‐5p, miR‐223‐5p and miR‐373‐3p. Furthermore, we included two additional potential biomarkers, miR‐3613‐3p and miR‐4529‐3p, based on our preliminary clinical study findings. To evaluate the expression levels of these miRNAs, we performed RT‐qPCR analysis on plasma samples obtained from healthy controls (HC) and BC patient, and compared the threshold cycle values (Ct). Information about the patients can be found in Table S2.

**FIGURE 6:**
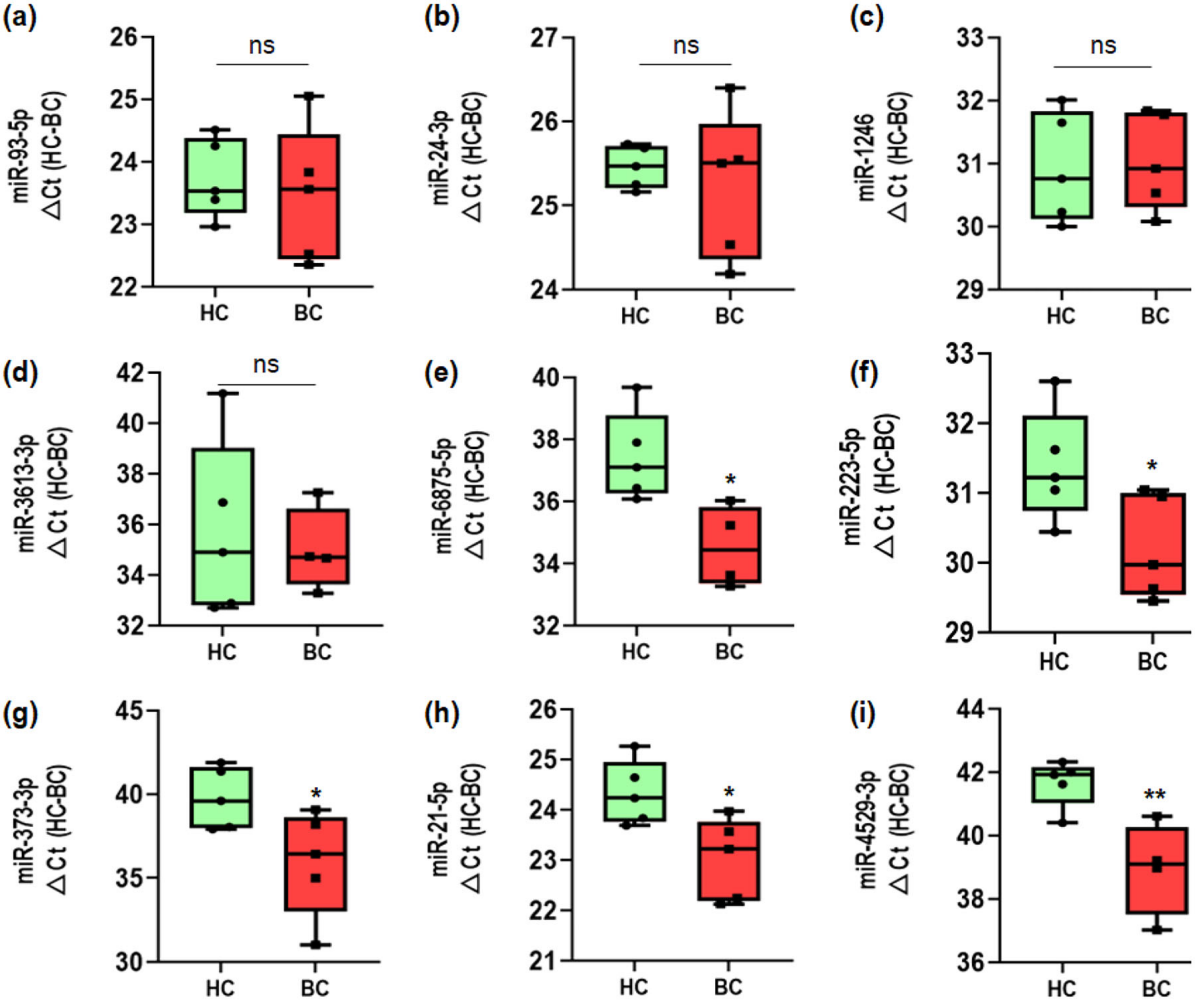
Relative expression levels of cancer‐related miRNAs in plasma EVs from healthy donors and patients with breast cancer using miRQuick. (a) miR‐93‐5p, (b) miR‐24‐3p, (c) miR‐1246, (d) miR‐3613‐3p, (e) miR‐6875‐5p, (f) miR‐223‐5p, (g) miR‐373‐3p, (h) miR‐21‐5p, (i) miR‐4529‐3p in plasma EV‐miRNA from healthy donors and patients with breast cancer analysed using RT‐qPCR and normalized (△Ct analysis) to healthy Ct value – patients Ct value. All values are presented as the median ± SD (*p < 0.05, *p* < 0.01, NS: no significance; *n* = 9–10).

Among the nine selected miRNAs, the first three miRNAs (miR‐93‐5p, miR‐24‐3p, and miR‐1246) selected from literature and one miRNA (miR‐3613‐3p) recommended from internal study did not show any significant differences between healthy and patient groups (Figure 6a–d). However, the remaining four miRNAs selected from literature (miR‐6875‐5p, miR‐223‐5p, miR‐373‐3p and miR‐21‐5p) and the one recommended from internal finding (miR‐4529‐3p) showed higher expression in BC (Figure 6e–i).

The expression of four miRNAs, namely miR‐6875‐5p, miR‐223‐5p, miR‐373‐3p and miR‐21‐5p, was found to be significantly increased in BC patients, which is consistent with previous studies (Jang et al., 2021; Shimomura et al., 2016; Yoshikawa et al., 2018). For instance, miR‐6875‐5p showed Ct values of 37.43 and 34.53 in healthy controls and BC patients, respectively, resulting in a ΔCt of 2.9, indicating a 7.5‐fold increase (=2^2.9^) in BC patients. This finding corroborates with a previous study that reported miR‐6875‐5p as an overexpressed biomarker in BC patients (Shimomura et al., 2016). Similarly, miR‐223‐5p and miR‐373‐3p, which were also found to be overexpressed in our study, have been reported as overexpressed miRNAs in BC in previous studies (Si et al., 2007; Yoshikawa et al., 2018). Furthermore, miR‐21‐5p, known to be involved in promoting angiogenesis and metastasis of cancer cells, has been recognized as an important biomarker not only in BC but also in other cancers (Gong et al., 2011; He et al., 2021; Petrović, 2016; Si et al., 2007). While information on miR‐4529‐3p in BC is limited, our internal findings suggest its potential as a diagnostic biomarker for BC, warranting further full‐scale clinical validation.

It may be worthwhile to re‐evaluate the potential of the four miRNAs that did not show significant differences as biomarkers. In BC tissues, miR‐93‐5p has been reported to be overexpressed, promoting angiogenesis by suppressing the expression of epithelial protein lost in neoplasm (EPLIN) (Liang et al., 2017). Similarly, miR‐24‐3p has been reported to be overexpressed in BC and promote cell proliferation by targeting p27Kip1 and suppressing cell death (Lu et al., 2015). Additionally, miR‐1246 has been found to be overexpressed in BC cells and inhibit Cyclin‐G2 (CGNG2) expression (Li et al., 2017; Zhang et al., 2020). Lastly, miR‐3613‐3p has been reported to be overexpressed in BC cells and promote cell proliferation and metastasis, making it a potential important biomarker (Liu et al., 2020).

However, in our study, we did not observe significant differences in the expression levels of miR‐93‐5p (*p* = 0.654), miR‐24‐3p (*p* = 0.599), miR‐1246 (*p* = 0.854) and miR‐3613‐3p (*p* = 0.716) between healthy controls (HC) and BC patients. It is important to consider that miRNA expression may vary among individuals and different types of carcinoma. For instance, miR‐24‐3p has been reported to decrease in expression level in metastatic cancer, inhibiting cell movement and invasion (Kang et al., 2017), suggesting the context‐dependent nature of miRNA expression in cancer. Further research and validation are warranted to fully assess the potential of these miRNAs as biomarkers in BC.

## 4 DISCUSSION AND CONCLUSION

We have demonstrated our newly developed method, miRQuick, for extracting miRNAs from samples in a single process using a charge‐interaction method, which addresses limitations of conventional methods, such as low yields and efficiency, and extended operation time. The improved efficiency of miRQuick can be attributed to the process sequence, which first involves aggregation‐based precipitation of native EVs followed by exosome lysis followed by nucleic acid extraction. For instance, the NucleoSpin kit utilizes protein precipitation by salting‐out with zinc chloride after adding lysis buffer to the sample, whereas miRQuick employs precipitation of clustered EVs prior to adding the lysis buffer to the sample. The sequence of processes appears to greatly influence the efficiency, depending on the type of sample. For urine samples, we observed more than a 10 Ct difference between NucleoSpin and miRQuick, highlighting the potential impact of process sequence on miRNA extraction efficiency.

While we have previously demonstrated the successful isolation of miRNAs using miRQuick, it is crucial to establish their origin as either from EVs or another source. To address this question, we conducted additional experiments wherein EV samples were subjected to RNase treatment, and the miRNA profiles were compared with those from the initial fluid. As depicted in Figure S3, the profiles of miRNAs extracted in both the presence and absence of RNase treatment exhibited nearly identical levels. These results strongly suggest that the majority of the miRNAs originate from EVs rather than circulating miRNAs in the bloodstream. Consequently, we have confirmed that the current miRQuick method effectively isolates EV‐derived miRNAs in a single‐step procedure.

Since the present miRQuick method based on charge‐based extraction does not selectively isolate EVs, proteins with negatively charged properties such as lipoproteins or fibrinogen can be co‐extracted with EVs. When these proteins exist as aggregates, they can often be counted as single particles in NTA measurements (Filipe et al., 2010). Indeed, SEM image analysis often observed material that appeared to be protein aggregates rather than EVs (Figure S3).

In accordance with the zeta potential analysis, it was observed that albumin and fibrinogen exhibited a negative charge, while γ‐globulin demonstrated a positive charge, as depicted in Figure 2b. Notably, the zeta potential of albumin (−14.79 mV) closely resembled that of EVs (−17.38 mV), prompting us to undertake further investigation of albumin following EV isolation. In the context of the Western blot analysis presented in Figure 4c, albumin exhibited distinct bands across all EV extraction methods, with variations in intensity among the methods. It is noteworthy that within the framework of this research, particularly utilizing the miRQuick method, the relative concentration of albumin in comparison to EV‐associated proteins was found to be the lowest compared to alternative techniques. Nonetheless, it is imperative to highlight that the majority of these residual impurities are effectively eliminated during subsequent washing steps, crucial for the isolation of miRNA. In the case of the miRQuick method, the EV lysis buffer, inclusive of proteinase‐K and surfactant, is meticulously formulated to facilitate the removal of undesired proteins during both the lysis step and subsequent washing processes. The aforementioned considerations collectively underscore the meticulous steps taken within the miRQuick methodology to ensure the selective enrichment of EV‐associated components, especially miRNA, while mitigating the presence of extraneous proteins.

Although the phenol–chloroform and spin‐column steps in the present method effectively remove undesired proteins, additional purification steps may be required to eliminate contaminants. However, these additional steps may result in a lowered yield of EV‐miRNA. Therefore, further improvements are necessary to enhance the specificity of EV isolation and minimize the presence of contaminating proteins.

In addition, we conducted a comprehensive analysis of particle size and scanning electron microscopy (SEM), which has substantiated our findings that previously clustered and precipitated EVs were effectively reconstituted into distinct entities through the elution process, leading to the formation of individual EVs. The elution process employed a typical buffer containing chloride ions (Cl^−^), a commonly utilized counterion in anion exchange chromatography due to its high charge density, enabling robust competition with other anions in the sample for binding to positively charged functional groups on the anion exchange resin. Complete resuspension as individual EVs may be challenging. However, when considering particle size and SEM analysis, it is evident that the majority of EVs were successfully resuspended into individual EV forms.

Furthermore, it is worth noting that PS is already recognized as a biologically compatible substance (Awotwe‐Otoo et al., 2012). As shown in Figures 4e and 4f, these results have unequivocally demonstrated that neither the elution buffer nor the PS affected the degradation of the biological activity of the isolated EVs (results not shown). Consequently, our method, which showcases both the successful resuspension of individual EVs and the preservation of their biological activity, holds significant promise for potential applications in the production of therapeutic EVs.

Through accelerated aging tests, PS solutions stored at 50°C showed the same EV isolation performance as those stored at 4°C or room temperature (23°C) for up to 11 weeks (Figure S4). These results indicate that the PS solution can be stored for 1 year at room temperature without refrigeration. In addition, PSs are very cost‐effective ($0.07/mg) compared to other cationic salts. For example, poly‐L‐lysine, which was used as a strong cationic polymer in previous studies, was developed for medical purposes and sold at a very high price (($200/mg), making it unsuitable for use as a general laboratory reagent. In addition, in the performance comparison test, the PS showed a 50% improvement. Therefore, the advantages of the miRQuick method are cost‐effectiveness as well as storage stability of reagents without cold chain storage (Devices, 2012).

In conclusion, we have successfully developed a convenient and straightforward method for isolating EV‐miRNA (miRQuick) that is well‐suited for liquid biopsy applications. MiRQuick demonstrates rapid and efficient recovery rates, outperforming other methods when working with plasma, urine and saliva samples. We have validated the efficacy of EV isolation using multiple techniques such as NTA, western blot and RT‐qPCR analysis. The charge‐based EV isolation method employed in miRQuick demonstrates remarkably high isolation efficiency, indicating its significant potential for practical applications. Furthermore, we have successfully extracted EV‐miRNA from BC patients, highlighting the potential for clinical implementation. Our method enables easy isolation of EV‐miRNA from liquid samples and holds promise for disease diagnosis, including BC.

## AUTHOR CONTRIBUTIONS

**Junsoo Park**: Conceptualization; formal analysis; investigation; methodology; visualization; writing—original draft. **Minju Bae**: Formal analysis; investigation; methodology. **Hyeonah Seong**: Formal analysis; investigation; methodology. **Jin hwa Hong**: Formal analysis; investigation; methodology. **Su Jin Kang**: Formal analysis; methodology; validation; visualization. **Kyung hwa Park**: Conceptualization; supervision; writing—review & editing. **Sehyun Shin**: Conceptualization; methodology; supervision; visualization; writing—review & editing.

## CONFLICT OF INTEREST STATEMENT

The authors declare no conflicts of interest.

## PATIENT CONSENT STATEMENT

Informed consent was obtained from the participant under the IRB guideline.

## Supporting information

Supporting Information

## Data Availability

The datasets analysed during the current study are available from the corresponding author on reasonable request.
