# Supplementary material for: An innovative charge‐based extracellular vesicle isolation method for highly efficient extraction of EV‐miRNAs from liquid samples: miRQuick
Source: J Extracell Biol. 2023 Dec 8;2(12):e126. doi: 10.1002/jex2.126 (PMC11080872; doi:10.1002/jex2.126)
Supplement: Supplementary file 1 — Supporting Information [file JEX2-2-e126-s001.docx]

**An innovative charge-based extracellular vesicle isolation method for highly efficient extraction of EV-miRNAs from liquid samples: miRQuick**

Junsoo Park^1,5^, Minju Bae^2^, Hyeonah Seong^2^, Jin hwa Hong^3^, Su Jin Kang^4^, Kyung hwa Park^3^*, Sehyun Shin^1,2,5^*

^1^ Department of Micro-Nano Engineering, Korea University, Seoul, 02841, Republic of Korea

^2^ School of Mechanical Engineering, Korea University, Seoul, 02841, Republic of Korea

^3^ Division of Oncology/Hematology, College of Medicine, Korea University, Seoul, 02841, Republic of Korea ^4^ Department of Bioengineering and Nano-Bioengineering, Incheon National University, Incheon 22012, Republic of Korea

^5^ Engineering Research Center for Biofluid Biopsy, Seoul, Korea

* To whom correspondence should be addressed:

Kyung hwa Park, MD, Ph.D.

Professor, College of Medicine, Korea University, Seoul, 02841, Republic of Korea E-mail: [khpark@korea.ac.kr](mailto:lexerdshin@korea.ac.kr)

Sehyun Shin, Ph.D. Professor, School of Mechanical Engineering, Korea University, Seoul 02841, Republic of Korea; Tel.: +82 2 3290 3377; Fax: +82 2 928 5825; E-mail: [lexerdshin@korea.ac.kr](mailto:lexerdshin@korea.ac.kr)

**Table S1. Stability of miRQuick demonstrated in an accelerated aging test**

| Ct value  (has-let-7a-5p) | 0 week | 1 week | 2 week | 4 week | 6 week | 8 week | 11 week |
| --- | --- | --- | --- | --- | --- | --- | --- |
| ExoQuick | 21.7 | 20.0 | 22.0 | 20.9 | 20.6 | 22.0 | 21.5 |
| miRQuick (RT) | 22.3 | 20.3 | 21.6 | 21.7 | 21.0 | 21.8 | 21.7 |
| miRQuick (4℃) | 22.3 | 19.5 | 21.3 | 21.4 | 20.9 | 21.6 | 21.5 |
| miRQuick (50℃) | 22.3 |  | 22.1 |  | 21.5 | 21.5 | 21.7 |

**Table 2. Table 2. Information on Clinical Trial Samples**

| **Sample ID** | **Gender** | **Age** | **Sampling Data** | **Subtype** |
| --- | --- | --- | --- | --- |
| BP295 | Female | 37 | 2020-09-24 | HER2 |
| BP246 | Female | 66 | 2020-09-24 | HR |
| BP213 | Female | 71 | 2020-09-24 | HR |
| BP308 | Female | 57 | 2020-10-12 | TPBC |
| BP361 | Female | 48 | 2020-10-12 | TNBC |
| NPF002 | Female | 29 | 2018-08-31 | Normal |
| NPF004 | Female | 32 | 2018-08-31 | Normal |
| NPF006 | Female | 27 | 2018-08-31 | Normal |
| NPF007 | Female | 26 | 2021-01-21 | Normal |
| NPF010 | Female | 37 | 2021-01-21 | Normal |


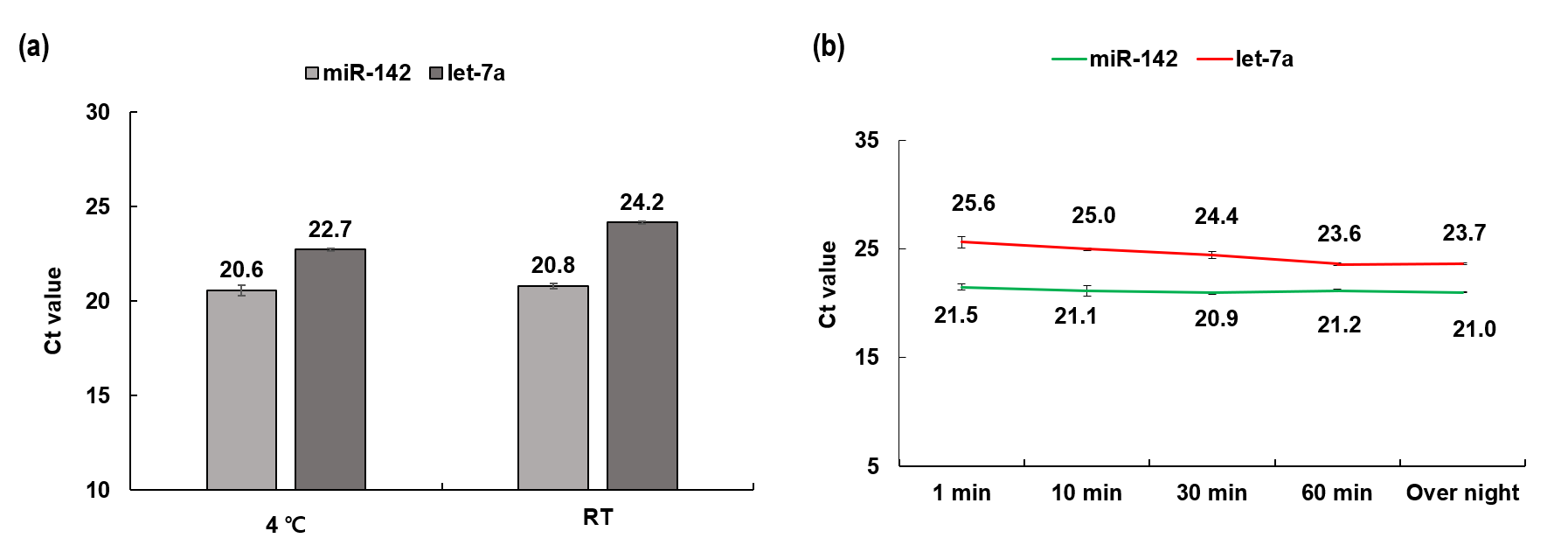


**Figure S1.** (a) EV-PSC incubation temperature. (b) Incubation time experiment at 4℃.


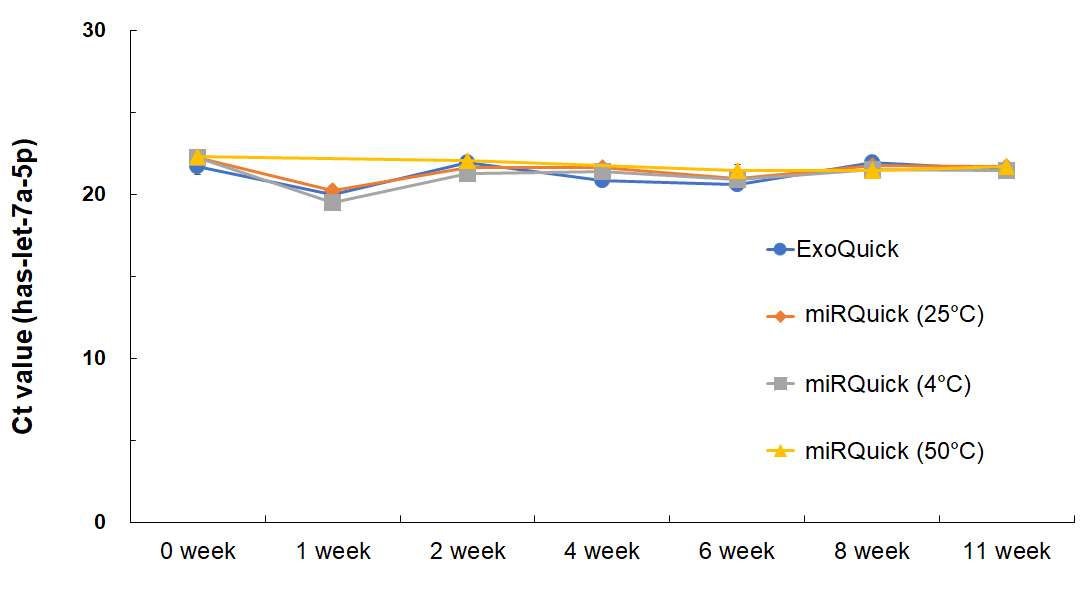


Figure S2. Protamine salt storage stability test. (a) EV-derived miRNAs (hsa-let-7a-5p) measured by Reverse Transcription-quantitative Polymerase Chain Reaction (RT-qPCR). (b) Ct value of the RT-qPCR results.

We used protamine salt for charge-based precipitation. To check the stability of the protamine salt solution, experiments were conducted at various temperatures based on the Arrhenius reaction rate function. By subjecting the solution to extreme environmental conditions, the data obtained from this experiment could be utilized for stability testing over an extended period. Our aim was to test the stability of the substance based on the precipitation efficiency of protamine salt. The commercial product ExoQuick was used as a control, and each protamine salt was tested at 4°C, 25°C, and 50°C over 11 weeks. The EV-derived miRNA, has-let-7a-5p, was measured through RT-qPCR, which allowed us to assess the EV precipitation efficiency. The results showed that even after 11 weeks, there was a difference of less than 1 Ct value at 4°C, 25°C, and 50°C. Applying this data to the Arrhenius reaction rate function (AAF = Q10[(TAA-TRT)/10]), we found that AAF = 5.7. (AAF= accelerated aging factor, TAA=Accelerated aging temperature, TRT=Ambient temperature, Q10=Rate of the chemical reaction, typically 2) This implies that the EV precipitation efficiency of the protamine salt solution was sustained over 439 days.


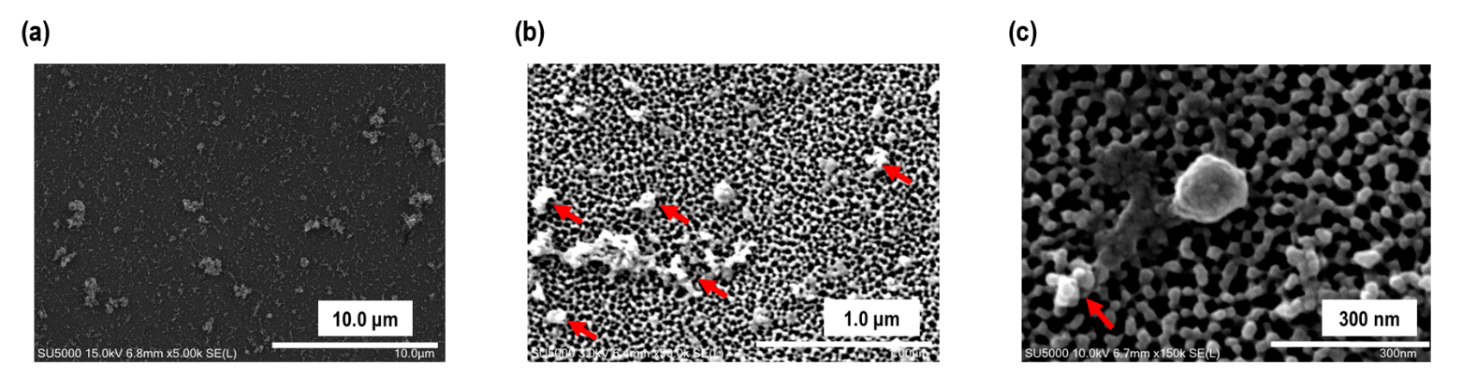


**Figure S3.** SEM image of isolated EVs using protamine salt precipitation from plasma sample. (a-c) SEM image of different ratio. Particles with shapes other than the spherical structure indicated by the red arrow are presumed to be non-EVs.


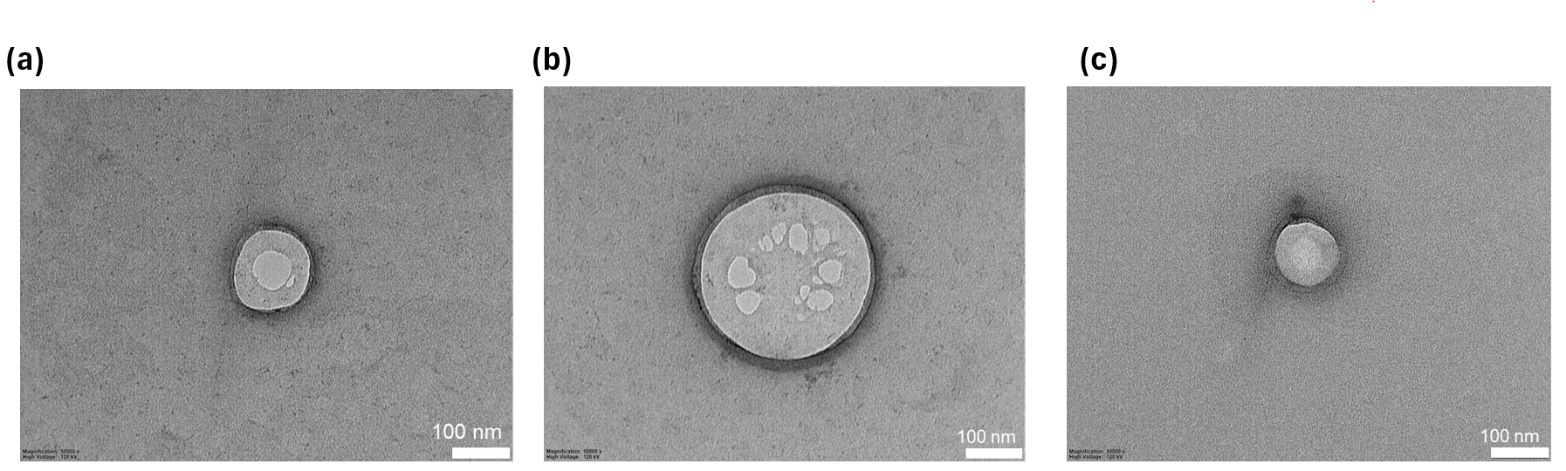


Figure S4 Characterization of Isolated EVs from Plasma, Saliva and Urine Samples. (a) ransmission Electron Microscopy image of isolated EV from plasma sample. (b) TEM image of isolated EV from a saliva sample. (c) TEM image of isolated EV from urine sample.


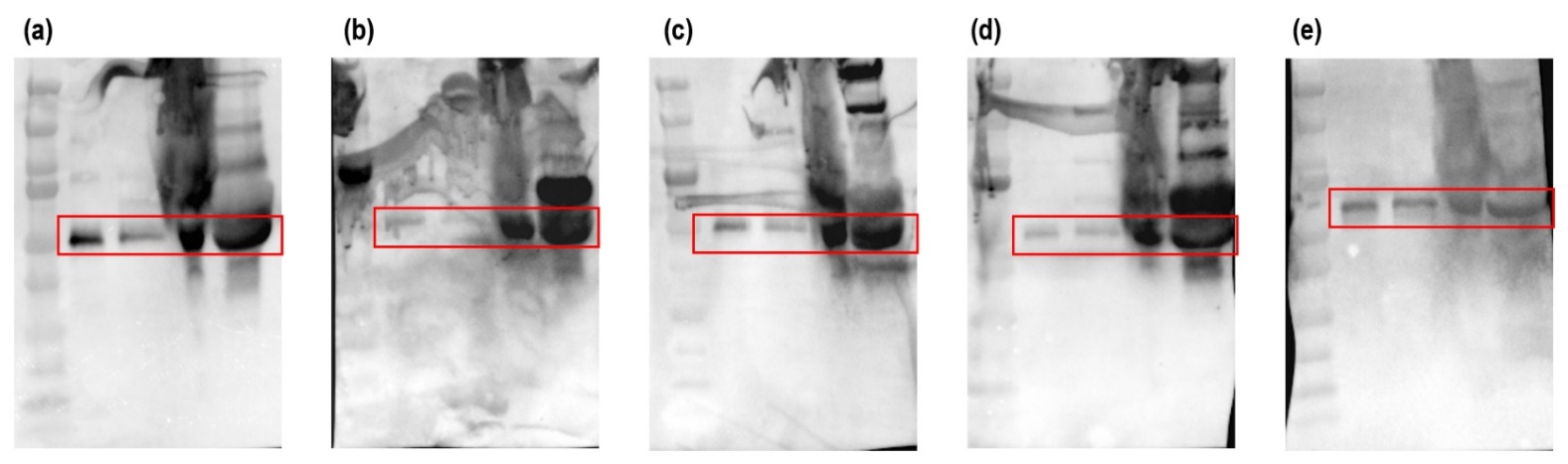

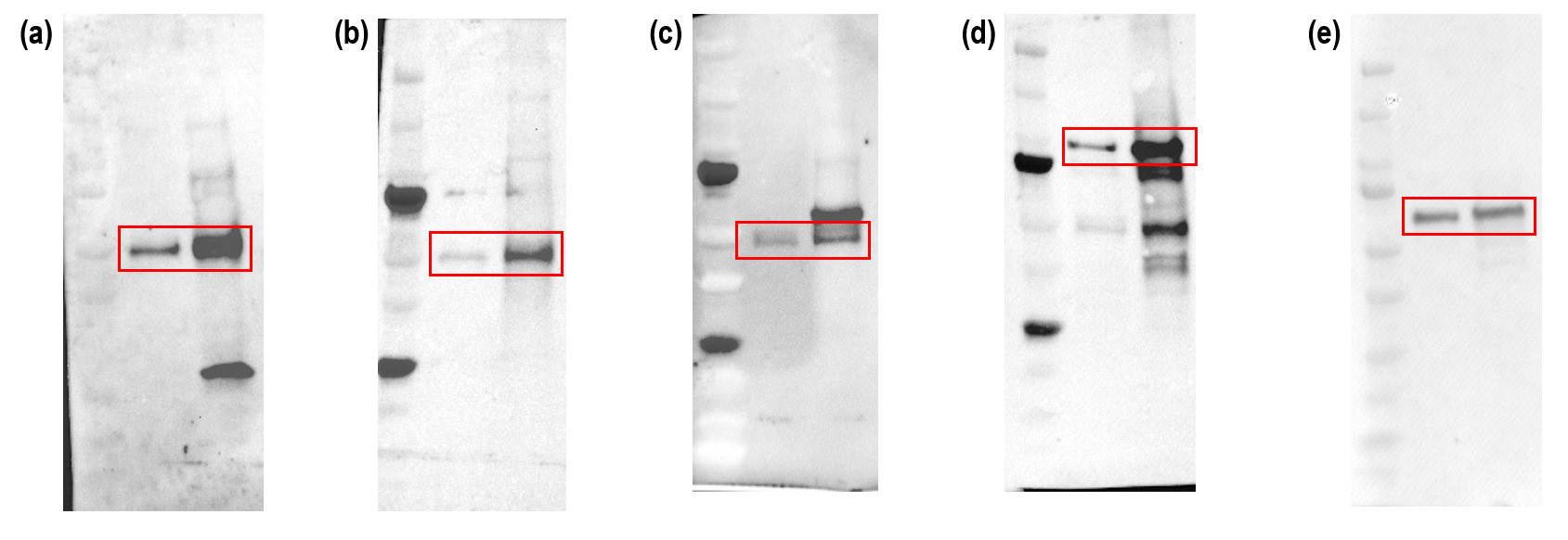


Figure S6. Whole western blot of Figure 4 There are five lanes, with ladder, UC, exoEasy, ExoQuick, and miRQuick from left to right, respectively. The red boxes indicate the positions of respective proteins. (a) CD9. (b) CD81. (c)TSG101. (d)Alix, (e)Albumin.

Figure S5. Whole western blot of Figure 3. There are three lanes, with ladder, urine, and saliva from left to right, respectively. The red boxes indicate the positions of respective proteins. (a) CD9. (b) CD81. (c)TSG101. (d)Alix, (e)Albumin.
